# A Dynamical Model of Genetic Networks for Cell Differentiation

**DOI:** 10.1371/journal.pone.0017703

**Published:** 2011-03-18

**Authors:** Marco Villani, Alessia Barbieri, Roberto Serra

**Affiliations:** 1 Modelling and Simulation Laboratory, Department of Communications and Economics, University of Modena and Reggio Emilia, Reggio Emilia, Italy; 2 European Centre for Living Technology, Venice, Italy; Ludwig-Maximilians University, Germany

## Abstract

A mathematical model is proposed which is able to describe the most important features of cell differentiation, without requiring specific detailed assumptions concerning the interactions which drive the phenomenon. On the contrary, cell differentiation is described here as an emergent property of a generic model of the underlying gene regulatory network, and it can therefore be applied to a variety of different organisms. The model points to a peculiar role of cellular noise in differentiation and leads to non trivial predictions which could be subject to experimental testing. Moreover, a single model proves able to describe several different phenomena observed in various differentiation processes.

## Introduction

A major challenge in complex systems biology is that of providing a general theoretical framework to describe the phenomena involved in cell differentiation, i.e. the process whereby stem cells, which can develop into different types, become progressively more specialized, The aim of this paper is indeed that of proposing a dynamical model of cell differentiation which is able to cover a broad spectrum of experimentally observed phenomena. The model we propose here is an abstract one (i.e. it does not refer to a specific organism or cell type) and it aims at describing the most relevant features of the differentiation process, which can be briefly summarized as follows:

1. different degrees of differentiation: totipotent stem cells can give rise to any cell type, typically undergoing some stages of progressive differentiation; there are also pluripotent and multipotent cells which can give rise to several, but not all, cell types;
2. stochastic differentiation: in some experimental conditions [1] [2] [3], both in vitro and in vivo, one can observe that a population of identical multipotent cells generates different cell types, in a stochastic way;
3. deterministic differentiation: in some experimental conditions (different from those of point 2 above), e.g. during embryo growth or in controlled experiments, specific signals trigger the development of a multipotent cell into a well-defined type [4], through a repeatable sequence of intermediate states. The signals correspond to the activation or deactivation of selected genes or groups of genes;
4. limited reversibility: the differentiation process is almost always irreversible (one-wayness) but there are limited exceptions, in that a cell which has reached an intermediate degree of differentiation can come back to a previous stage, under the action of appropriate signals [5] [6];
5. induced pluripotency: it has been observed that also fully differentiated cells can come back to a pluripotent state by modifying the expression level of some genes [7] [8];
6. induced change of cell type: it has been observed also that the expression of few transcription factors can convert one cell type into another, e.g. mouse fibroblasts into induced functional neurons [9].

Since cell differentiation is tightly related to the activation/deactivation of groups of genes, it is appropriate to look at models of gene networks in order to describe the dynamics of differentiation.

Note that the presence in the same system of properties 2 and 3 implies an intriguing mixture of stochasticity and determinism. Therefore it is not obvious that a single model can describe all these phenomena. There are indeed models of differentiation which are able to describe some of them [3] [10] [11]; they make use of a continuum description and, in part because of computational limitations, are bound to take into account the contributions of only few genes. Here we hypothesize that the robust properties of differentiation are rather the outcome of the interaction of very many genes, so our model is based on a simplified dynamical model of genetic regulatory networks, namely noisy random Boolean networks (NRBNs for short), which actually allow simulations of large networks [12]. NRBNs represent an extension of the well-known model of random Boolean networks [13] [14] [15] [16] (RBNs) that, in spite of their approximations, have been able to describe important experimental facts concerning gene expression[17] [18] [19].

A classical RBN is a dynamical system, based on a directed graph with N nodes (genes), which can assume binary values 0 or 1 (inactive/active); time is discrete, with synchronous updating of all the node values. Each node has exactly 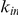 input connection; in the classical model used here 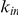 is the same for all nodes and the input are chosen randomly with uniform probability among the remaining N-1 nodes (prohibiting multiple connections). To each node a Boolean function is associated, which determines its value at time 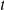 from the values of its inputs at the previous time step. The Boolean functions are chosen at random for every node, by assigning to each set of input values the outcome 1 with probability 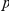. Both the topology and the Boolean function associated to each node do not change in time. The network dynamics is discrete and synchronous, so fixed points and cycles are the only possible asymptotic states in finite networks. Extensive studies have shown that, considering the robustness with respect to small changes in initial conditions it is possible to distinguish different dynamical regimes: ordered, critical and disordered (often called “chaotic” although, since it refers to cycles, the term pseudo-chaotic would be more appropriate). In the ordered regime small transient perturbations die out, while in the disordered one they initially tend to grow. Networks whose structural parameters take values intermediate between those which are typical of ordered and disordered ones are called critical.The interested reader is referred to existing excellent reviews for a more complete discussion of RBNs [13] [14] [15] [16].

The most interesting behaviour has been shown by nets in a critical regime, which show both robustness and adaptiveness [20] and it has been suggested that living organisms are driven by evolution in a critical dynamical state (at or close to the boundary between ordered and chaotic phases) [21] [22]. Recent results, which compare RBN simulations with experimental data, lend support to the view that biological genetic regulatory networks indeed operate close to the critical region [17] [18] [19]. Therefore in the present study all the results shown refer to RBNs whose parameters lie in the critical region (in particular, we choose for all the simulations the values 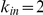 and 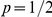).

**Figure 1:**
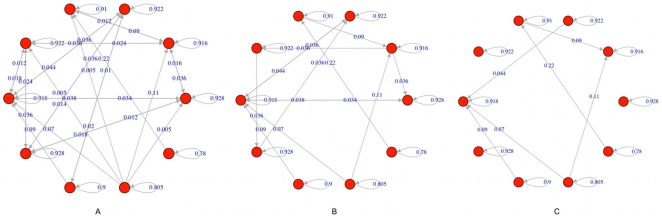
Attractor transition graphs in a RBN. Circles represent network attractors; arrows represent transitions among attractors induced by single spin flips. All the nodes of all the states of each attractor are perturbed one by one; the numbers on each arrow are the fractions of cases where the corresponding transition is observed, so they provide an estimate of the probability that, by flipping at random the state of a node in an attractor, that transition takes place. In (a) the complete attractor transition graph is shown, while in (b) and (c) only those links which correspond to above-threshold transitions are retained (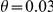 in (b) and 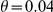 in (c)).

Note however that attractors of RBNs are unstable with respect to noise even at low levels. Consider for example a transient flip of a randomly chosen node when the system is in a state of one of its attractors: even if the flip lasts for a single time step one sometimes observes transitions from that attractor to another one (see Figure 1a).

Such transitions are observed in almost all the networks, and their frequency scales with the network size in a way described in [12]. In particular, the probability that a flip on a randomly chosen node leads the system to a different state cycle decreases with the network size, but in a sublinear way, so that the overall number of transitions turns out to be an increasing function of N.

Noise is known to play a role in key cellular processes [23] [24] [25] [26] [27] [28], and it has been proposed since the seminal work of Kupiec [29] that it be involved in differentiation; random fluctuations also play a role in some existing mathematical models of differentiation [11] [30].

We will therefore investigate the asymptotic dynamics of the network subject to noise, modelled by the transient flip of a randomly chosen node which lasts for a single time step; after that, the node follows the rules of the network deterministic dynamics. This is indeed the smallest possible random fluctuation affecting a Boolean system. It will also be assumed that the noise level is small enough to allow the system to relax to an attractor before a new flip occurs. Several simulations have indeed shown that, while the transient from a random initial state to an attractor may be long, the transitions between two different attractors almost always require a small number of steps. This hypothesis allows one to make use of the knowledge of the attractors of the deterministic system to analyze the behaviour of its noisy version, thereby strongly simplifying the description of the asymptotic dynamics of the stochastic system [31].

It would be natural to identify the attractors of RBNs with cell types, as originally proposed by Kauffman [14] [15] [16], since they correspond to different coherent dynamical states of activation, with the same genome (i.e. topology and Boolean functions). However, since attractors (this term will always be used here for those of the deterministic system) are unstable with respect to noise, they can no longer be associated to cell types. A possible way out was proposed by Ribeiro and Kauffman [31] who observed that there exist sets of attractors, which they called ergodic sets, which entrap the system in the long time limit, so the system continues to jump between attractors which belong to the set. It would then be natural to associate cell types to such ergodic sets, but unfortunately it turns out that most NRBNs have just one such set (at most 2 of them have been observed in extensive simulations). This strong limitation on the number of ergodic sets rules out the possibility to associate them to cell types.

A possible solution to this problem was proposed in [12] and is briefly summarized in the next section, where it is also shown that by a proper interpretation it can describe in an elegant way the fact that there exist different degrees of differentiation, and that it provides a natural way to simulate stochastic differentiation (i.e. properties 1 and 2). In the following section we show that the same model describes also deterministic differentiation, when appropriate signals are provided (i.e. property 3), while in a further section we show that it also accounts for limited reversibility, induced pluripotency and induced change of cell type (properties 4, 5 and 6). Finally, in the last section we discuss the biological meaning of the key hypotheses, the implications of the model and possible experimental tests.

## Results

### Threshold ergodic sets and stochastic differentiation

Observe that the kind of noise which is taken here into account is fairly intense, as it amounts to silencing an expressed gene or to express a gene which would otherwise be inactive; therefore it is an event which is much less frequent than, e.g., molecular-scale fluctuations. Consider now the case where the transition between two attractors occurs only when a single specific node is flipped. This may well be an event too rare to happen with significant probability in the cell lifetime. Therefore we will introduce a threshold, and will take into account only those transitions that may happen by a number of flips above that threshold (Figure 1b and Figure 1c). Note that here we are not considering multiple flips (these would be even rarer) but different paths that lead from one attractor to another. It is intuitively clear that the threshold should be related to the level of noise in the cell, and it has indeed been shown elsewhere [12] that it scales with the reciprocal of the frequency of flips, i.e. the noise level. A more thorough discussion of the biological significance of the threshold will be deferred to the final section.

Since we consider only above-threshold transitions, the notion of Ergodic Set, precisely defined in [12] and [31], has to be modified in that of a Threshold Ergodic Set, that is a set of attractors that entrap the system in the long time limit, so the system continues to jump between attractors belonging to the set.

Formally, let 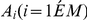 be the M attractors of a given network (under the action of the deterministic transition functions), and let A be the set of such attractors. We say that an attractor 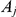 is directly 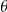-reachable from another attractor 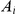 if at least a fraction 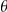 of different flips leads the system, when it is in attractor 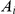, to attractor 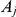. We also say that 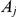 is indirectly 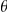-reachable from 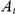 if there exists a path which leads from 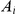 to 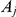 via transitions between pairs of attractors which are directly 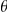-reachable.

A Threshold Ergodic Set (briefly, TES or, when the value of the threshold is considered, 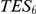) is defined as a subset of A composed by attractors which have the following properties:

- any member of the 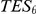 is 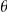-reachable from any other member of the set, not necessarily in a single step;
- given that threshold value, no transition can make the system leave the 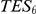

Within this definition, we can describe an ergodic set as a 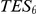 with 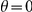.

**Figure 2:**
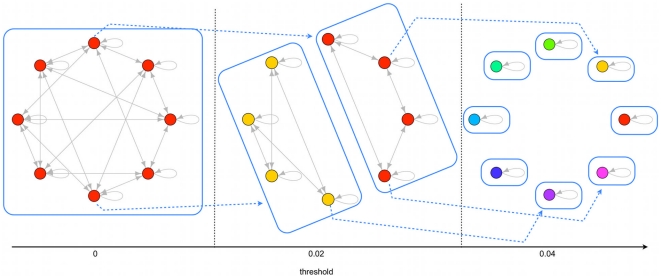
TESs and stochastic differentiation. As the threshold is increased the single 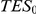 breaks into smaller disjoint TESs, which correspond to more differentiated cells, until eventually final cell types are reached (i.e. single-TESs). Stochastic differentiation is explained by the fact that the new TES which is reached when the threshold is increased depends upon the attractor in which the cell is found and upon the node which is flipped (a few possible transitions are shown by dotted lines).

Let us now consider what happens by gradually increasing the threshold. At 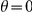 one typically has a unique TES but, by increasing the threshold, it breaks into some disjoint TESs. By further increasing the threshold these TESs in turn break into smaller ones until, at high enough levels of the threshold, all attractors are also TESs (i.e. they cannot be abandoned). The process is shown in Figure 2. The ratio between the total number of TESs and the total number of attractors increases as the threshold is increased, and for each network there is a value such that, when 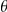 exceeds that value, all the attractors are also TESs. A quantitative analysis of the way in which the number of different TESs increases as a function of the threshold, for different network sizes, can be found in [12].

We propose to associate cell types to TESs. They represent indeed coherent stable ways of functioning of the same genome (i.e. connections and Boolean functions) even in the presence of noise. The problem that hampered the straightforward association of cell types to ergodic sets is no longer present in this case, since there may be several TESs in the same network.

The degree of differentiation is supposed to be related to the possibility for the cell, in its asymptotic state, to wander in a portion of phase space which should be smaller for a more differentiated cell. In the present framework, a convenient proxy for the available portion of phase space is the number of attractors belonging to the TES. Therefore, a totipotent cell should be associated to the 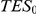 (i.e. the one found when 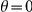), while as the threshold is increased more differentiated forms appear (pluripotent or multipotent cells), corresponding to smaller TESs like those shown in Figure 2. At high enough threshold values all the attractors are TESs, and these should describe the fully differentiated cells. A TES with a single attractor will be called a single-TES, while a TES with two or more attractors is a multi-TES.

In order to describe differentiation, in the present framework it is assumed that it implies a change in the threshold, which in turn implies a change in the noise level. Differentiation increases if the threshold increases, i.e. the noise level decreases, and this latter effect could be related to an improvement in the mechanisms whereby fluctuations are kept under control [27]. The association of differentiation to changes in the threshold level represents the most stringent outcome of this model, and is in principle amenable to experimental test, as it will be discussed in the final section. For the time being let it suffice to note that association of differentiation to different levels of noise has already been proposed on theoretical and experimental bases [30] [32] [33] and that a higher noise level in undifferentiated cells, with respect to more differentiated forms, has been actually reported [34] [35] [36].

While the above hypothesis explains in a straightforward way the fact that there are different degrees of differentiation (i.e. property 1), related to different threshold values, it should be noted that also stochastic differentiation [34] [37] (property 2) is described by the model. Indeed, the fate of a given cell depends on the particular attractor where it is found at the moment when the threshold is increased: the new type will be the one described by the TES to which that attractor belongs, at the higher threshold value (see Figure 2).

Note that the above framework allows one also to understand important experimental findings [2] where it has been observed that a population of monoclonal partially differentiated cells actually hosts a rather wide distribution of concentrations of some molecular markers. By selecting and isolating, from the initial population, a subpopulation with similar values of these markers, it turns out that the initial wide distribution is eventually restored. It is apparent that this behaviour is entirely coherent with the picture where each cell, at a given time t, is in an attractor of a TES: if a subpopulation, composed by cells in the same attractor, is picked up, the whole TES is recreated under the action of noise. Similar results have also been obtained with embryonic stem cells [32]. Note also that the experimentally observed subpopulations show different patterns of gene expression, some of them being close to that of the cells which are reached after differentiation has taken place; this again is what one could expect from the TES picture. It is also worth noticing that the authors of this study report that the kinetics of the process is coherent with a picture of a cell switching between different metastable attractors, like it has been supposed here. The cells considered in this study can differentiate to different fates, but it has been observed that by chemically stimulating them with erythropoietin one always obtains the same cell type: this is a nice example of deterministic differentiation, which will be discussed in the next section.

### Switch nodes determine the cell fate

There exist indeed several processes, e.g. during the embryogenesis, in which cell differentiation is not stochastic but it is driven towards precise, repeatable types by specific chemical signals, which activate or silence some genes. These signals are thus represented in the model by permanent perturbations of a node (for reasons of simplicity we will consider the fixing of the value of a single node at a time), which fix its state to 1 or 0. In order to describe these deterministic differentiation processes in our model we couple these permanent perturbations with an increase of the threshold (which by itself would lead to the stochastic differentiation shown in Figure 2).

The model will be considered able to describe deterministic (signal-driven) differentiation if one can demonstrate the existence of switch genes, whose permanent activation or inhibition always leads the system through the same differentiation pathway, i.e. nodes that uniquely determine to which TES the system will evolve. Switches are precisely defined as follows: starting from a certain TES, if fixing the value of a node from all phases of each attractor of that TES the system goes always in the same attractor (when the threshold is increased), then the perturbed node is a switch (in that TES). A less stringent yet meaningful definition could be given by requiring that the perturbation leads the system to attractors belonging to the same TES; the present one is however easier to verify. The existence of switch nodes has actually been verified to be a widespread property (found in about 1/3 of the nets), thereby proving the effectiveness of the model. Note that it is not necessary to prove that switches exist for all the NRBNs, it is indeed sufficient to show that they are present in a significant fraction of them, so that natural selection can pick up the “good” ones.

**Figure 3:**
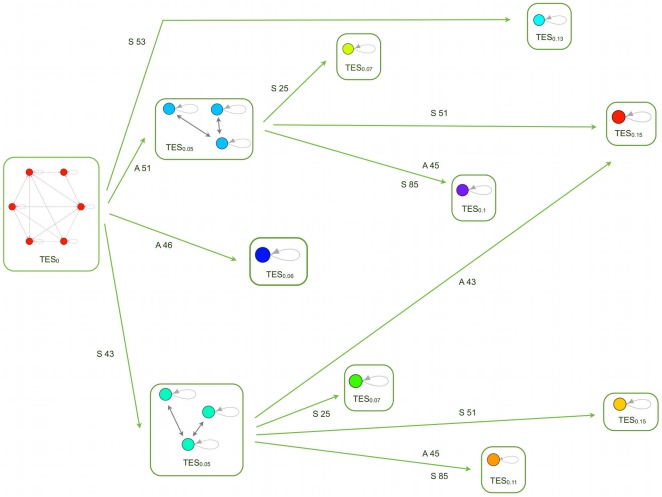
A case of deterministic differentiation. In this schematization each box represents a TES and each circle represents an attractor. Arrows indicate possible different path differentiation and labels on arrow indicate the switch: the number is the number of the node that act as a switch, A means that it is switched-on and S means that it is switched-off. Note that it is here possible to observe two kinds of redundancy: in one case a particular TES can be reached acting on different switches of the same multi-TES (as shown by double labels on the same arrow); in the other case the same TES can be reached acting on switches belonging to different multi-TESs (i.e. the same TES can be reached from different pathway), as in the case of the red single-TES, which can be reached either from the azure or from the turquoise multi-TES.

In Figure 3 one can see an example of differentiation, from a multi-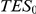 to a set of single-TESs, which shows a remarkable qualitative similarity with differentiation diagrams of real cell lineages, like e.g. hemopoietic cells.

Some considerations arise from the experiments we performed: first of all, this case represents just one possible diagram obtained from simulations; the system shows indeed a very rich and complex landscape of possible behaviours, as in biological differentiation. It is interesting to observe that two types of redundancies have been observed in real cells, when moving in a signal-driven way from an intermediate type A to a more differentiated type B: in some cases the transition can be achieved by acting on different genes of A [38], and in other cases B may be reached also by acting on a cell type C which does not belong to the lineage of A [23] [39]. These correspond in the model respectively to the cases in which a new TES can be reached from the same multi-TES acting on different switches and to the cases in which a single type can be reached from different pathways. Both can be actually observed in the example shown in Figure 3.

It is also important to observe that the model accounts in a straightforward way for differentiation diagrams where there are both deterministic and stochastic steps. A similar combination has also been observed in nature, e.g. in hemopoietic cell differentiation [40].

Note also that a chemical signal can be modelled by the permanent perturbation of a gene, also when the latter is not a switch. In this case, by definition, if the threshold is increased one observes differentiation towards different TESs: therefore according to the model one can observe stochastic differentiation (albeit of a different type) even when chemical signals are present; a statement which can be subject to a experimental verification.

### Simulating induced pluripotency and other properties

In recent years considerable attention has been raised by the discovery of induced pluripotency (property 5) where overexpression of a few transcription factors (from 1 to 4) in differentiated cells can make them “come back” to a less differentiated state [7] [8] [41]. Simulating such a process of dedifferentiation by a decrease of the threshold would be straightforward but, since there is no evidence that such a process actually takes place in experiments, we checked whether dedifferentiation can be achieved without modifying the threshold, by simply fixing the value of a gene to 1 permanently so to simulate its overexpression (of course this makes sense on those genes which are not always active in the model).

**Figure 4:**
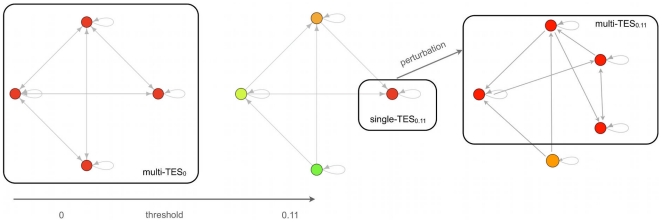
Yamanaka-like in silico experiment. Schematization of the Yamanaka experiment: starting with one multi-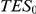 (the leftmost graph), which represent a totipotent stem cell, one increases the threshold until a single-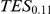 is reached (composed by the rightmost attractor of the central graph), which represents a fully differentiated cell. Then, by permanently perturbing a node belonging to the single-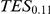 to the fixed state 1 (overexpression) one obtains the multi-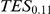 (the rightmost graph). This graph refers to a network with 10 nodes.

This phenomenon can actually be observed in some networks, as shown in Figure 4. This behaviour is not generic, and it is found rarely, but also in biological systems there are just a few genes that can give rise to induced pluripotency. Note also from Figure 4 that most of the attractors of the 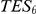 reached in this way are identical (apart from the perturbed node) to those of the original 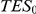, a situation which can be summarized by saying that the two TESs are similar to each other - and this closely parallels what has been experimentally observed. Note also that the above description belongs to the set of so-called stochastic models of iPSC that seem in accordance with known experimental facts [42].

**Figure 5:**
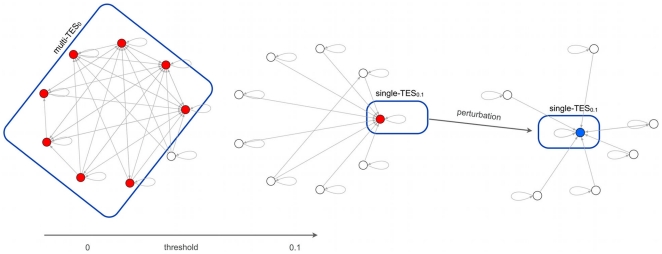
An example of transition between differentiated cells. By increasing the threshold to 0.1, from the initial multi-TES0 a single-TES is reached which represents a fully differentiated cell type. A permanent perturbation leads to a different attractor which, at the same threshold level, is also a single-TES, corresponding to a different cell type.

**Figure 6:**
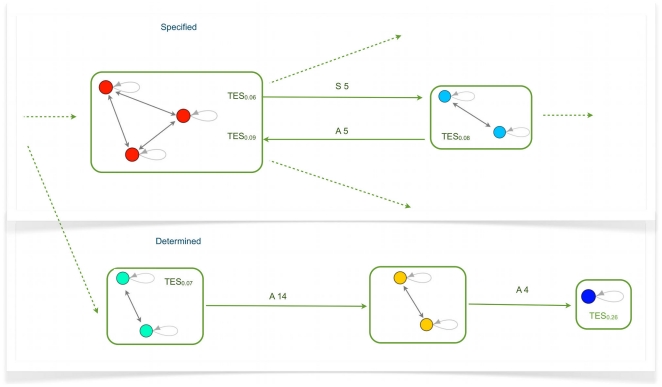
Examples of specified and determined cell. An example of exception to irreversibility is shown in the upper box (which is part of a larger differentiation graph) where one sees that by inhibiting a specific switch node the red TES differentiates to the azure one, but the path can be reversed. The lower box describes another (irreversible) branch of the same network.

Finally, it is important to observe that the model is actually able to describe also property 6, i.e. possible transitions between two differentiated cell types (as shown in Figure 5), as well as property 4, concerning the existence of limited exceptions to the irreversibility of cell differentiation, as shown in Figure 6. Note that the difference with respect to induced pluripotency (Figure 4) is that in the present case the return to a less differentiated state can be accompanied by an increase in the threshold, while in simulating the Yamanaka experiment no change of the threshold was performed.

## Discussion

The most interesting features of the model presented here can be briefly summarized as follows:

1. a single model describes all the main features of differentiation (listed at the beginning of the Introduction);
2. the explanation of differentiation makes use of the global properties of a generic dynamical system, without resorting to detailed hypotheses concerning very specific control circuits, so differentiation is linked to sets of attractors of a large network, rather than to a specific interactions between few genes. Note also there is no need to introduce epigenetic barriers [3];
3. the control of the noise level plays a crucial role in differentiation; while a role for noise in differentiation has already been hypothesized [30] [32] [33], in our system it is indeed the control of the noise level that drives differentiation (to the best of our knowledge a similar hypothesis has been proposed only by Hoffman et al; their model is however different in many respects from ours, and in particular it requires a number of assumptions concerning the effect of the environment on the cellular noise and on the proliferation rate);
4. switches provides an elegant way to model deterministic differentiation, without requiring further ad hoc assumptions.

Note that some care must be exercised in applying our results to present-day biological genetic networks, which are likely not randomly wired, but have been shaped by evolution. So it may well be that present-day differentiation pathways are controlled at least in part in a more rigid and perhaps reliable way, but even in this case our model should hold as a proposal for the origin of differentiation, and it should also provide a partial description of modern differentiation. The most striking result obtained here concerns the importance of the threshold: if we permanently modify the expression of one or a few genes without acting on the threshold, the breakup of a TES into smaller disjoint ones has not been observed. This statement is in principle subject to experimental testing, provided that we define the biological meaning of the threshold. As it has been repeatedly stressed, this could be related to the level of noise in the cell [12].

If the threshold is related to the noise level, and if differentiation requires a change of the threshold, then differentiation should be accompanied by a change in the noise level. It is important to remark that flips (active/inactive) similar to those adopted here have actually been observed [43] as well as to make reference to some works which suggest that in stem cells more genes are usually active than in differentiated ones, albeit at a lower level [34] [35]. Since this entails a smaller number of copies of m-RNA molecules per cell, and since the relative role of fluctuations is higher when the number of exemplars is lower, this indicates that noise can indeed be higher in stem cells than in differentiated ones. It is also particularly interesting to observe that it has recently been reported [2] [35] [36] that the state of gene expression levels of (at least some) stem cells can be described as slowly itinerating among several quasi-stable states, a description which fits that of a TES.

The deterministic differentiation processes which are observed e.g. in embryo growth require that the threshold of a cell can change when needed. It is natural to suppose that the threshold itself is under genetic control, so that it can be modified when appropriate. Among the various mechanisms, which may be involved in such control, let us mention that *i)* the folding/unfolding of chromatin can modify the level of noise of many genes [44] and *ii)* the production of miRNA can silence genes which are expressed at low levels, thereby making expression noise vanish [45]. These two mechanisms can suppress noise around the inactive state of the genes. Other mechanisms can be at work to stabilize the active state, for example by producing more copies of m-RNA per unit time [46], by reducing the degradation rate of the proteins or by using buffer circuits to keep constant nonzero activation values [45].

On the theoretical side, there are several aspects that are worth exploring, including those concerning the generality of our results. The general picture of the cell as a dynamical system, and the idea that differentiated cells are more constrained in their wandering in phase space can be applied also to other models of gene and cell dynamics [10], and the question can be raised concerning the possibility of obtaining similar results also with these other gene network models. We have modelled here only a single cell, lumping the effect of the other neighboring cells in a “signal” which sets the value of a particular gene; it would be interesting to explore along these lines also the role of the interactions among communicating cells in differentiation.

In the present version of the model, the threshold level is modified by in an exogenous way but, as it has been observed, it is likely to be itself under genetic control. It would therefore be interesting to develop a model where the threshold itself is ruled by a pattern of activation of some genes, and look at the unfolding of differentiation. A limited step in this direction was performed in [47] where the effects of mutations in particular genes (threshold regulating genes) were analyzed. Other research directions include the use of variations of the classical RBN model, motivated by increasing knowledge of the actual properties of biological systems (like e.g. scale-free networks, modular networks, different updating schemes, multiple-valued or continuous models, etc.).

Let us finally remark that the availability of sophisticated system-level models like this can lead to a deeper understanding of the process and can provide impulse to the experiments by suggesting testable hypotheses, in particular those concerning the importance of controlling the noise level in differentiation.

## Materials and Methods

The simulations concerning RBNs were made using a software developed in house, written in C++. Different network sizes were tested, unless otherwise stated, the results shown in this paper refer to networks with 100 nodes (a few smaller networks with 10 and 20 nodes were also simulated, as well as some larger ones with 200 nodes).

Except for the 10-node and 20-node networks, exhaustive testing of the possible initial conditions is impossible, so in networks of 100 or more nodes attractors were found starting by 10.000 randomly chosen initial conditions.

In all the simulations the number of incoming links is 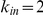 and the bias of the Boolean function is 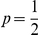, thereby guranteeing that our networks sare critical according to the definition recalled in Section 1 and thoroughly discussed in [14] and [16].

The search was performed with an algorithm able to find attractors with periods not larger than 500 time steps (and a maximum transient of 1000 steps). It turns out that these search parameters allow one to find an attractor for all the initial conditions in about 99% of the random networks.

The transition graph between different attractors was obtained by perturbing (independently) each node of each state of each attractor. For each perturbation the new attractor was found, thereby determining the weights of the links of the attractor transition graph.

The search for TESs was made using a software developed in house, written in C++. The algorithm was based on the search for the strongly connected components of the attractor transition graph (taking into account the level of the threshold). For each strongly connected component it was then checked whether it actually entrapped the system, a necessary condition for it to be a TES.

The results concerning the switches have been obtained as follows, starting from critical RBNs with 100 nodes. In order to describe cells with the same genome, i.e. the same structure of the RBN, which can evolve to different fates we limited our analysis to networks with more than one switch and where there are at least two switches leading to different asymptotic states. Starting from TES0 we searched for a switch and, when we found one, we fixed its value and grew up the threshold to obtain a 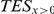 composed by a smaller number of attractors. Then we repeated the procedure starting from the newly found 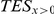 to find a 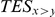 with an even smaller number of attractors, until we found a single-TES (i.e. a fully differentiated cell). In this way we explored just one of the possible paths, only a tree branch, so in order to obtain a complete picture of the possible fates we iterated the procedure for all the branches of the root (the initial multi-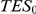) and all possible sub-branches. Eventually we found all the possible system fates, which can be represented e.g. as in Figure 2.

The software codes used for simulation of the dynamics of RBNs, for searching the attractors, for determining the attractor transition graph and for finding the TESs are available upon request by one of us (A.B. email: alessia.barbieri@unimore.it).
